# The Awareness Framework: A Novel Approach for Understanding HIV Testing and Disclosure in HIV-discordant Dyads

**DOI:** 10.4172/jaa.1000057

**Published:** 2013-02-06

**Authors:** Nora E. Rosenberg, Audrey E. Pettifor, William C. Miller

**Affiliations:** 1 UNC Project, Lilongwe, Malawi; 2 Department of Epidemiology, University of North Carolina, USA; 3 Department of Medicine, University of North Carolina, Chapel Hill, USA

## Introduction

HIV testing and counseling (HTC) is rapidly being brought to scale in sub-Saharan Africa [1]. Scale-up has been driven primarily by the goal of linking HIV-infected persons to treatment. But what impact will HTC scale-up have on HIV prevention, especially as new biomedical HIV prevention interventions are introduced?

Consistent with the *Couples HIV Testing and Counseling Guidelines* recently released by the World Health Organization, we assert that the prevention impact of HTC will hinge on whether both members of HIV-discordant dyads receive HTC and whether they share their HIV status with each other. However, a better understanding of the prevention impact of other awareness possibilities is needed. We introduce a novel framework for considering a person’s awareness of his/her own HIV status (through HTC) and his/her partner’s HIV status (through HIV disclosure) within HIV-discordant dyads. This framework is useful for understanding HTC trends, examining behavioral and biomedical risk in partnerships, and ultimately optimizing the impact of HIV prevention.

## Describing the Awareness Framework

HIV awareness within dyads involves two stages–testing and disclosure. In the first stage, persons can learn their own HIV status through HIV testing. In the second stage, they can inform their sex partners of their HIV status through disclosure. Within this framework, HIV testing is a prerequisite for disclosure. In couples HTC, the two stages typically occur simultaneously in both partners. With most other HTC strategies, clients are encouraged to disclose to sex partners, but there is no guarantee that this will occur.

For prevention purposes, these two stages of awareness must be considered within HIV-discordant dyads. HIV-discordant dyads account for all sexual transmission of HIV. We use the phrase “HIV-discordant dyad” broadly to refer to all sexual contacts with one HIV-infected and one HIV-uninfected person. These dyads can be homosexual or heterosexual, married or unmarried, long-standing or brief. We distinguish the term “HIV-discordant dyad”, from the more commonly used terms HIV-discordant “couple” or “partnership” which typically refers to a subset of dyads who are in long-term, stable, and often marital or cohabiting relationships.

**Figure 1:**
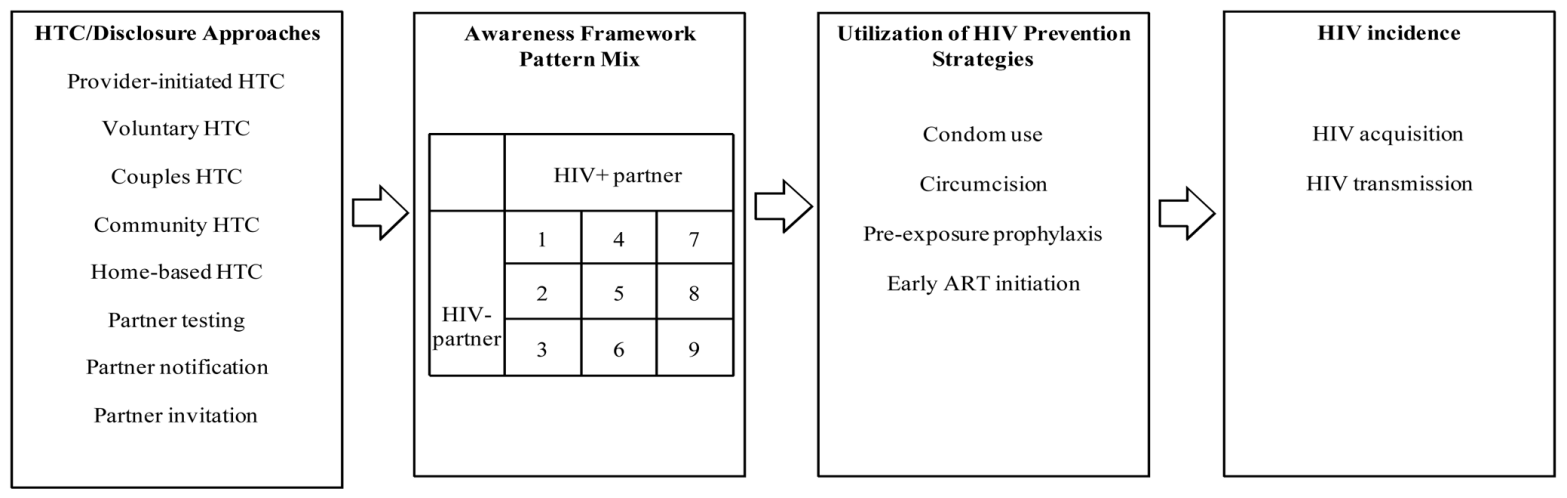
The Role of the Awareness Framework in HIV Prevention The Awareness Framework is likely to be an important mediator of all HIV prevention. Different HTC and disclosure modalities will lead to different Awareness Framework pattern mixes. In turn, these pattern mixes are likely to impact the utilization of different HIV prevention strategies, including condoms, circumcision, pre-exposure prophylaxis, and early ART initiation. These strategies have been shown to lower the probability of HIV transmission or acquisition.

When HIV testing and disclosure in each member of the dyad are considered jointly, nine “awareness patterns” are possible (Figure 1). In one extreme pattern, neither partner has been tested for HIV (Table 1, pattern 1). In the other extreme pattern, both partners have been tested for HIV and have mutually disclosed their HIV status to each other (Table 1, pattern 9). In an intermediate pattern, both partners have been tested individually but neither has disclosed to the other person (Table 1, pattern 5). Six additional patterns reflect other combinations of testing and disclosure - two in which only one partner has been tested but has not disclosed (Table 1, patterns 2 and 4), two in which one only partner has been tested and has disclosed (Table 1, patterns 3 and 7), and two in which both partners have been tested, but only one has disclosed (Table 1, patterns 6 and 8). Several patterns, such as neither partner having ever been tested (Table 1, pattern 1), have been described. Other patterns, such as table 1, pattern 6 or 8, where both partners have been tested, but only one has disclosed, have been overlooked.

Within an HIV-discordant dyad these patterns may change over time. For some dyads, mutual awareness (Table 1, pattern 9) may occur soon after the relationship forms. For other dyads, it may take years to progress to mutual awareness. And for others still, mutual awareness may never occur.

The Awareness Framework is likely a mediator of HIV prevention, as depicted in figure 1. Different HTC approaches, such as individual versus couple or client-initiated versus provider-initiated will likely result in different distributions of Awareness Framework patterns (i.e. pattern mixes). In turn, these pattern mixes are likely to affect the use of prevention strategies, including condoms, circumcision, pre-exposure prophylaxis (PrEP), and early antiretroviral therapy (ART) initiation, which reduce HIV transmission [2,3] or acquisition [3–8].

## HTC and Disclosure Approaches

The first set of questions to consider with the Awareness Framework is how different HTC and disclosure approaches impact the HIV-discordant dyad pattern mix. Both the type and scale of these approaches are important.

Different HTC approaches result in different distributions of Awareness Framework patterns. For example, client-initiated voluntary counseling and testing (VCT) models, which tend to be time consuming and counseling-intensive, often result in higher rates of disclosure, but reach fewer people. On the other hand, provider-initiated testing and counseling (PITC) models, which tend to be brief with minimal counseling, may result in lower rates of disclosure but reach more people. The shift from VCT to PITC approaches may result in more persons aware of their own HIV status (e.g. Table 1, pattern 5), but a lower proportion who are mutually aware (e.g. Table 1, pattern 9).

Several HTC approaches are explicitly dyad-oriented, and designed to achieve higher rates of disclosure than VCT or PITC. Couple’s HTC has been implemented in stand-alone VCT settings [9], antenatal clinics [10], and home-based care [11], leading to most dyads being mutually aware (Table 1, pattern 9). Intensive counselor-facilitated disclosure [12], invitations for male partners [13], provider-based partner notification [14,15], and partner testing [16] are also designed to yield high rates of disclosure by both partners. Although such strategies are more costly, they could prove cost-effective in the long-term, once their prevention impacts are considered.

## Awareness Framework Pattern Mix

A second set of questions to address with the Awareness Framework is the prevalence of each pattern. Without this basic step it is not possible to determine which patterns are riskiest. Although all nine Awareness Framework patterns have never been characterized, individual self-report offers some insight into the pattern mix. In most African countries, before HIV treatment was available, very few persons had ever been tested, suggesting most dyads were in table 1, pattern 1 (neither partner tested) [17–19]. Currently, larger shares of populations have been tested and in some settings a large proportion report disclosure to sex partners [20–23], suggesting a much broader distribution of patterns. However, even in a setting where 75% of persons have been tested and 75% of these persons have disclosed, only about a third of HIV-discordant dyads would be expected to have mutually tested and disclosed (Table 1, pattern 9)^1^.

Characterizing the pattern mix is possible in national or population-based surveys with questions on individual testing and disclosure, the ability to link dyads together, and HIV status. These characterizations would be an important step to understanding the pattern mix at a point in time, and its evolution over time.

## Utilization of HIV Prevention Strategies

A third set of questions to assess with the Awareness Framework is how each pattern affects use of different HIV prevention strategies, including condoms, pre-exposure prophylaxis, circumcision and early ART initiation.

We hypothesize that table 1, pattern 9, in which both partners have been tested and disclosed, is the most protective for a range of prevention behaviors. In this pattern, both partners are aware that the HIV-uninfected partner is at risk for HIV acquisition. They can make decisions individually or together to protect the HIV-uninfected partner. However, the relative transmission risk within the other eight patterns is less straightforward. In each, the presence of risk within the dyad is uncertain for at least one partner. For example, for a table 1, pattern 5 dyad, even though both persons have been tested, neither has disclosed and therefore neither is aware that the dyad is HIV-discordant. Similarly, in table 1, patterns 3 or 7, when the status of one dyad member is known to both partners, they may assume incorrectly that the other dyad member has the same HIV status.

## Condom use

The combination of HTC and mutual disclosure is known to have a strong impact on condom use. HIV-discordant dyads testing together (Table 1, pattern 9), report dramatic increases in condom use, [24,25] and display lower HIV incidence rates [3,26–28]. However, the association of each of the other eight patterns with consistent condom use is less clear because most studies have compared individuals’ behavior in one *set* of patterns to individuals’ behavior in a different set of patterns. For example, HIV-infected persons who know their own HIV status (Table 1, patterns 4–9) tend to report much higher levels of condom use than HIV-infected persons who do not know their HIV status (Table 1, patterns 1–3) [24,25,29]. Such comparisons make it appear that all persons in table 1, patterns 4–9 have equal risk and that all persons in table 1, patterns 1–3 have equal risk, when in fact the risk within each set of patterns may vary considerably. Similarly, disclosure by HIV infected persons is often, though not always, associated with increased condom use [20,30,31], but this also has not been studied by pattern. Separate comparisons of each pattern (1–8) to pattern 9 would provide a clearer picture of risk.

## New biomedical HIV prevention strategies

HTC will surely play a role in access to new biomedical prevention strategies. Only HIV-uninfected persons who have been tested (Table 1, patterns 2, 3, 5, 6, 8 or 9) will be able to access pre-exposure prophylaxis, or male circumcision. Similarly, only HIV-infected persons who have been tested (Table 1, patterns 4–9) will be able to access early ART initiation for “treatment as prevention.” Clearly, HTC scale-up is essential for biomedical prevention.

Mutual disclosure of HIV status may also play an important role in biomedical prevention. Just as pattern 9 has been associated with higher adherence to condoms, it is likely to be associated with higher adherence to PreP and early ART initiation [32,33]. The groundbreaking HPTN 052 trial of early ART initiation by HIV-infected persons provides an important example of this possibility [2]. This trial was conducted among mutually aware HIV-discordant dyads, all in pattern 9. In this trial not only did 95% of couples report consistent condom use, but adherence to ART was very high. When early ART initiation is implemented elsewhere, some persons taking ART will be in pattern 9 dyads, but others will undoubtedly be in pattern 4–8 dyads. HIV-infected persons in pattern 4–8 dyads may be less likely to use condoms and may face partner-level barriers to adherence. As a result, effectiveness might be undermined.

The Awareness Framework may also help explain different efficacy results in the PrEP trials. In both FEM-PrEP and Partners PrEP, participants were taking oral doses of FTC/TDF daily. But in the FEM-PrEP trial, HIV-uninfected women enrolled as individuals and could have been in any of several dyad patterns (2,3,5,6,8, or 9) or in HIV-concordant-negative dyads. In contrast, in the Partners PrEP study all participants were in mutually aware HIV-discordant dyads (Table 1, pattern 9). In FEM-PrEP, adherence was poor and PrEP was not efficacious. In contrast, in Partners PrEP, adherence to PreP was excellent [34], and acquisition was reduced by 75% [8]. The different pattern likely explain differences in adherence and ultimately to differences in efficacy.

## Discussion

The Awareness Framework offers two novel contributions to thinking about HIV prevention. First, it adds nuance to the discussion about HIV status awareness within HIV-discordant dyads. Although, many have advocated for couple-based strategies for Africa, none have delineated all of the eight possible alternatives to mutual awareness, even though some may be quite prevalent and meaningful. Second, the Awareness Framework informs thinking about how different HTC modalities may translate into utilization of many HIV prevention strategies and ultimately different prevention effectiveness.

The Awareness Framework has real-world relevance for HTC programs. The types of HTC that are implemented will affect the Awareness Framework patterns which could have a profound impact on use of proven biomedical HIV prevention interventions. Assessing the shift in the Awareness Framework patterns over time will identify the gaps in testing or disclosure and in HIV-infected or HIV-uninfected persons. A better understanding of these gaps is necessary for determining how best to direct resources.

The Awareness Framework is a simple representation of the patterns of testing and disclosure within dyads. It is not a conceptual model for understanding why people seek HTC or why they disclose. It also does not address whether all means of achieving a certain pattern are equally effective. Certainly, the nine patterns are not the only factors underlying sexual risk-taking in partnerships. Gender, dyad stability, substance use, intimate partner violence, age, age gaps, and sexual communication are other key factors that are undoubtedly critical within dyads.

Additionally, although HIV-discordant dyads are the primary unit of analysis within the framework, understanding how these dyads relate to the sexual networks they are a part of is essential. HIV-discordant dyads in riskier patterns will have a larger impact on HIV incidence if they are engaging in higher levels of concurrency or have more central positions in sexual networks.

Overall the Awareness Framework provides a more nuanced consideration of HIV testing and disclosure, which underlies all HIV prevention. Ignoring the complexity of testing and disclosure relationships within dyads may lead to oversimplified understandings of HIV prevention, suboptimal HTC strategies, and missed HIV prevention opportunities.

## Figures and Tables

**Table 1:** The Awareness Framework - Nine Patterns for HIV- Discordant Dyads There are nine possible awareness patterns within HIV-discordant dyads. The HIV-infected person may or may not have been tested, and if tested, may or may not have disclosed. Similarly, the HIV-uninfected person may or may not have been tested, and if tested, may or may not have disclosed. Understanding how each of the nine patterns is associated with uptake of and adherence to different HIV prevention strategies is important.

*HIV-uninfected partner*	*HIV-infected partner*		
	Has not tested for HIV, has not disclosed	Has tested for HIV, has not disclosed	Has tested for HIV, has disclosed
**Has not tested for HIV, has not disclosed**	1 HIV-infected not tested, HIV-uninfected not tested	4 HIV-infected tested, HIV-uninfected not tested	7 HIV-infected disclosed, HIV-uninfected not tested
**Has tested for HIV, has not disclosed**	2 HIV-infected not tested, HIV-uninfected tested	5 HIV-infected tested, HIV-uninfected tested	8 HIV-infected disclosed, HIV-uninfected tested
**Has tested for HIV, has disclosed**	3 HIV-infected not tested, HIV-uninfected disclosed	6 HIV-infected tested, HIV-uninfected disclosed	9 HIV-infected disclosed, HIV-uninfected disclosed

## Footnotes

1 If 75% of persons know their own HIV status and 75% of these persons disclose, then 56% of persons are likely to have tested and disclosed (0.75×0.75). The probability that both persons have tested and disclosed is 32% (0.56×0.56). This assumes testing and disclosure in the two partners are independent.
